# Use of Planning Training Courses and Activities to Enhance the Understanding of Eco-Community Planning Concepts in Participatory Planning Workshop Participants: A Case Study in Taiwan

**DOI:** 10.3390/ijerph16091666

**Published:** 2019-05-13

**Authors:** Chen-Yi Sun, Hsiu-Hsiung Tai, Ai-Ching Yen

**Affiliations:** Department of Land Economics, National Chengchi University, Taipei 11605, Taiwan; hhtai@nccu.edu.tw (H.-H.T.); irene50604a@gmail.com (A.-C.Y.)

**Keywords:** citizen participation, sustainable development goals, co-design, co-planning, indigenous

## Abstract

In recent years, in order to make community planning content closer to people’s life needs and psychological expectations, and to obtain the support of the people, “citizen participatory planning” and “community-engagement” have become two important strategies of the community planning process. In this study, an indigenous people participatory planning workshop was conducted with the support of government funds, and pre-training and post-training questionnaires were completed by the participants of the planning training of the citizen participation planning. Through questionnaire analysis, this study obtained data of the participants’ cognitive status related to community planning and analyzed the basic background of the participants in order to determine the effectiveness of the planning training. According to the results of this study, most of the participating citizens had a basic understanding of the “community environment”, “the relationship between ecological knowledge and community planning”, and “community identity” before the training. Moreover, the research results also confirm that planning training can effectively enhance participants’ understanding of community planning, spatial planning, planning tools, planning laws, and the environment of the community. Additionally, planning training also contributes to the implementation of participatory decision-making and the promotion of public support for planning content. However, it is necessary to obtain a more comprehensive understanding of the needs of participants, and to make appropriate adjustments to the planning training courses and activities in order to obtain stable training effectiveness and build the basic ability of citizens with respect to participatory planning.

## 1. Introduction

In 2014, all 193 United Nations member states proposed a set of 17 Sustainable Development Goals (SDGs) as reference goals for the international development community. The United Nations had promoted the Millennium Development Goals (MDGs) for many years before the SDGs were announced [1]. In order to explore the leading role and impact of SDGs on sustainable development, there have been many studies on SDGs since 2014 [2,3,4,5]. In order to explore ways for Taiwan to achieve sustainable development, there have been many researches on sustainable cities, ecological communities, and green buildings. These studies provide relevant strategies, assessment tools, methods, and policies for the sustainable development of Taiwan [6,7,8,9,10,11,12,13,14,15,16,17]. In order to develop sustainable cities and communities, one of the SDGs, it is a very important task for governments to plan and maintain a livable and sustainable community.

According to the results of relevant research literature, the planning of sustainable communities must protect residents’ lives and preserve economic production and the ecological environment [18,19,20]. Citizen participation is a very important part of ensuring the practicality of eco-community planning [7,18,19]. After the establishment of the sustainable community, the government must try to re-align policies and incentives to complement the existing social capital network formation to optimize local organizations’ access to outside economic and human resources [7,21,22,23,24]. This research focuses on how to establish the most important foundation for a sustainable and eco-community through citizen participation to improve the efficiency and sustainability of eco-community planning in the planning and design phase.

In recent years, in order to improve the efficiency of urban and community planning works and achieve the goal of sustainable management, the Taiwanese government has required that planners must appropriately incorporate citizen participation in the planning process. For example, in the procedures related to urban planning and urban renewal, a "public hearing" must be held to understand the opinions of the public, and in the relevant meeting, "stakeholders" are allowed to attend and allow suggestions in advance. This approach not only helps planners to understand the real needs of the citizens, but also enables the citizens to understand the main themes and ideals of eco-community planning through the citizen participation activities, and additionally lets the planning decision content receive the support of the citizens. Furthermore, when public participation mechanisms are integrated into disaster management planning and community planning, it also helps to mitigate community disasters [25].

After the citizen participation has been incorporated into the community planning process, there are still many problems to be resolved. A study summed up 9 obstacles to implementing community participation [26]: (1) the paternalistic role of development professionals; (2) the inhibiting and prescriptive role of the state; (3) the over-reporting of development successes; (4) selective participation; (5) hard-issue bias; (6) conflicting interest groups within end-beneficiary; (7) gate-keeping by local elites; (8) excessive pressure for immediate results: the accentuation of product at the expense of process, and (9) the lack of public interest in becoming involved. Moreover, the study also proposed 12 recommendations for community participation, one of which was to respect the indigenous community’s contribution as manifested in their knowledge, skills, and potential.

Additionally, the cultural diversity of the participants’ backgrounds brings some challenges to the practice of citizen participatory planning. However, the participatory planning process can also help to bridge the gap between outside planners and community occupants, and enhances community planning awareness and consensus [27]. In order to take advantage of participatory planning, planners and policymakers should broaden the definition of “public” in citizen participation, include other stakeholders in the broader urban development process, and put citizens before plans in order to give them a real chance to offer some opinions [28].

Participatory planning can increase the effectiveness and adaptivity of the planning process. Besides, citizen participation can be the essential element in making the planning process a learning system [29]. However, since they have often never been educated in planning and design, people do not have basic knowledge of these concepts. Therefore, in the participatory planning process, it is easy to cause misunderstanding or poor communication efficiency between residents and professional planners due to a lack of consensus and common language. However, this issue may be eliminated through planning training and planning tools.

In the relevant research cases, through the use of activity arrangements, and planning tools (drawing, model making, and interactive games), participants can fully express their opinions and learn the basic knowledge of planning and design in the process of participatory planning [30]. Basically, participatory planning forms an integral part of future planning and place-making processes, a socially constructed process which can shape cities by capital investment designed to generate economic growth and promote cultural tourism. Therefore, planners must stress the importance of the creative participatory processes to attract stakeholders and enhance their willingness to partake in the participatory planning processes, as well as to identify creative participatory planning tools that can be used to enhance participatory planning within the place-making process [31].

Relevant research results confirm that, in order to enhance the effectiveness of participatory planning and design, the inclusion of digital tools in planning helps to increase participants’ understanding of the plan [32]. Geographic information systems (GISs) have the function of describing basic information in the planning process. In particular, when the new tool entitled Bottom-Up GIS (BUGIS) is used in participatory planning activities, it helps planners to understand the local residents’ perceptions, such as what is liked, disliked, or desired about a given locale [33]. Another paper is devoted to exploring the potential contribution of Participatory GIS (PGIS) to participatory spatial planning and summarizing the potential of participatory geographic information tools; meanwhile, it’s results indicate that in future participatory planning, participatory mobile GIS, participative three-dimensional modeling, and visualization features, these three technologies (GIS, BUGIS, and PGIS) have a high potential for application [34].

In summary, citizen participatory planning (including planning learning, training and activity) could be a great help for sustainable and eco-community planning. Therefore, the main purpose of this study is to clarify whether planning training is effective in enhancing the community’s awareness, basic planning knowledge, and understanding of planning. The results of this study can be used not only as a criterion for planners to carry out participatory planning related activities in the future, but also as a reference for the government to formulate policies of citizen participatory planning.

## 2. Materials and Methods

Planning training could be the important first step in order to help participants building up their basic skills of planning discussion and planning operations. This study uses planning training courses and activities to enable citizens to learn the basic planning concept, planning logic, and basic drawing ability to promote the understanding of the operation mechanism of spatial planning, commonly used planning tools, and related legal knowledge. In the traditional planning process, the government and professional planners often have insufficient understanding of the culture, history, and life patterns of the local people. In particular, when many participants in the citizen participatory planning are indigenous people, for planners, more understanding of the life needs and cultural background of the Indigenous People through the participation process should be very important for planning tasks. The current lifestyle of Taiwan’s indigenous community is not much different from that of the average person, but most of the indigenous community still retain some of their traditional culture, family systems and ceremonies. Therefore, based on the lifestyle, cognition and culture that continue the tradition, the views of the indigenous community on the content of community planning are still slightly different from the average person.

In addition, citizens lack relative knowledge of map interpretation, spatial analysis, planning tools, and other basic planning knowledge. Therefore, the planners and citizens always have problems of communication due to the lack of common language. Therefore, in order to enhance the effectiveness of participatory planning, planning training courses and activities can help governments and planners understand citizens’ living habits and the concept of community environment.

After the training by the actual investigation, data analysis, topic discussion, preliminary design, and other processes, citizens can effectively communicate with the planners when expressing the ideal of planning and design. When the planning-related materials are gradually collected and analyzed, citizens can express and interpret their own spatial experience based on traditional knowledge of land. Additionally, the participants can also use planning tools to specifically show the value of the public’s knowledge of the natural environment.

With the above foundations, citizens and planners can further enter the stage of joint planning. After discussing the development vision, clarifying the needs of life, and understanding the dilemmas and challenges, the citizens and planners will jointly develop specific plans. Eventually, after the initial completion of the planning content, a consensus meeting will be held for discussion, feedback on various opinions will be obtained, and, in the process of revision and consensus-seeking, the plan blueprint supported by the majority will finally be obtained. Therefore, it is very important to let the public get the basic knowledge and concepts of planning through appropriate workshops and courses before participatory planning.

**Figure 1:**
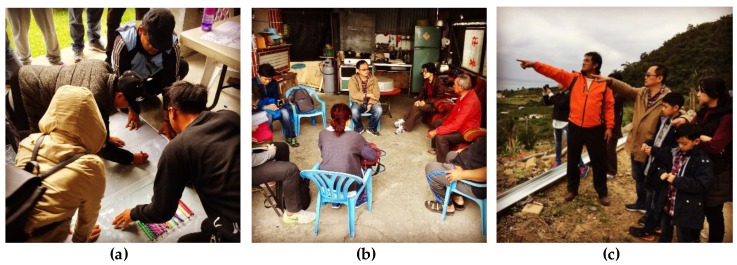
Photograph of participatory planning activities in this case study. (**a**) community environment and historical exploration activities; (**b**) senior citizen consultation meeting; (**c**) community environmental survey and exploration.

Through the participatory planning workshop, citizens can not only gradually establish the basic cognition and ability to plan their community and environment, but also apply traditional land managements, space utilizations, and lifestyles. Therefore, this study handled the participatory planning workshop of indigenous community planning with government support in 2017. This workshop lasted a total of 12.5 hours (Table 1) including a few courses and activities. Participants in this workshop were from more than 70 indigenous communities. Some participants were recommended by local governments and some were self-registered. In this workshop, the main content is divided into three parts: (1) planning theory; (2) planning tools and training; and (3) practice and discussion. In addition to the lectures, there were three participatory planning activities in this workshop (Figure 1): (a) community environment and historical exploration activities; (b) senior citizen consultation meeting; (c) community environmental survey and exploration. The main purpose of this workshop was to train a group of citizens as intermediaries to act as facilitators for future participatory community planning process.

Through training courses and related activities, the workshop achieved the goal of training the citizens of the indigenous community to have the basic ability to participate in planning. During the workshop activities, participants were asked to fill out a feedback questionnaire (Table 2) to determine the effectiveness of training courses and activities through the analysis of the differences in the basic planning cognition. The questionnaire for this study was self-evaluated by participants before and after the training. Before the course, the participants gave a score from 1 to 10 for each basic planning cognition, and immediately gave basic planning cognition a training achievement score of 1 to 10 after the completion of each stage of the workshop. The results of the questionnaire analysis in this study can help planners to understand the participant-training effectiveness of the courses and activities.

## 3. Results and Discussions

### 3.1. Participants’ Basic Information

This study used the complete questionnaire data obtained from the Participatory Planning Workshop to facilitate the analysis of training effectiveness. There was a total of 167 participants (Table 3), including eight indigenous tribes and Han nationalities (non-indigenous) in Taiwan. Most of the participants were aged between 20 and 70 years old, four were over 70 years old, and four did not fill in the age question. More than half of the participants lived in the tribe for more than 50% of the time and belonged to the group with close relatives in real life and tribal planning. Most of the participants had college or higher education background, with 45 participants having master’s or doctoral degrees. About half of participants are leaders of the community or the cadres of community organizations, and more than half of participants have more than two years of community involvement.

As far as the overall data are concerned, the indigenous people who participated in the planning training courses and activities generally had a higher basic understanding of the “community environment” and “community identity”. Indigenous participants also had a certain level of basic understanding of “community planning”, “the relationship between aboriginal ecological knowledge and community planning”, and “the vision of the community’s planning”. However, participants’ basic understandings of the three aspects of “spatial planning”, “planning laws and regulations”, and “GIS, GPS, and spatial analysis” were low. Additionally, after the planning training, the ability of indigenous participants was greatly improved in various cognitions. Participants’ cognitive improvement was most obvious in the three aspects of “spatial planning”, “planning laws and regulations”, and “GIS, GPS, and spatial analysis”.

### 3.2. Differences in Training Effectiveness among Participants of Different Background

#### 3.2.1. Ages

**Figure 2:**
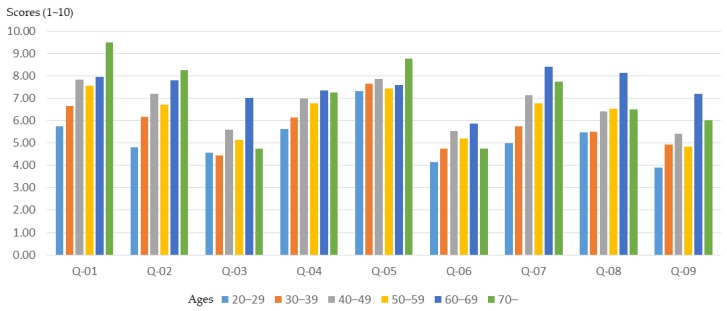
Workshop participants’ basic understanding before training, for different age groups.

**Figure 3:**
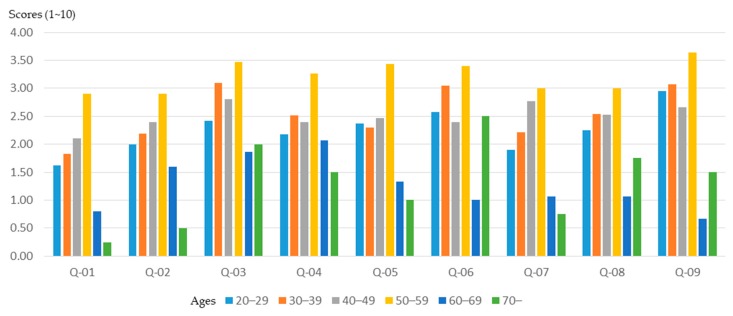
Basic cognitive growth of workshop participants after training, for different age groups.

**Figure 4:**
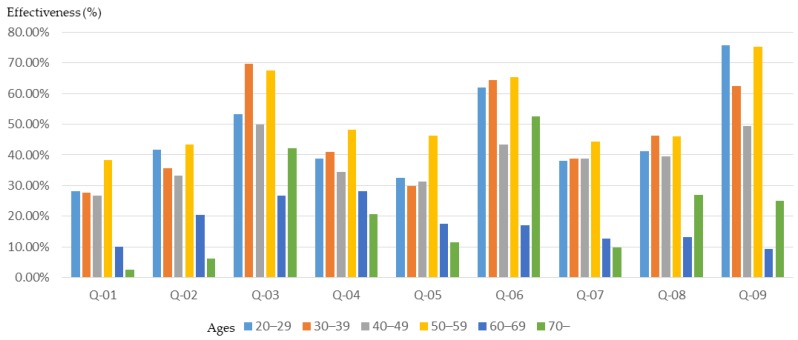
Basic cognitive growth rate of workshop participants after training, for different age groups.

The age difference of the participants in the workshop will not only cause differences in the basic planning understanding, but also in training outcomes. In order to clarify the relationship between the age of participants and training outcomes, this study conducted a relevant analysis. The results (Figure 2) show that participants with higher ages (over 60 years old) had a higher initial understanding of planning, especially in the “understanding of the community environment”, “understanding of community planning”, and “the sense of community identity”. However, compared to younger participants (20–49 years old), participants in the middle-aged class (50–59 years old) have become more proficient in planning-related concepts and understanding after training in the planning course (Figure 3 and Figure 4).

After the planning training, participants’ understanding of planning concepts increased, especially the “understanding of spatial planning”, “understanding of planning laws and regulations”, and “understanding of GIS, GPS, and spatial analysis”. However, although older participants (over 60 years of age) had a higher initial understanding of planning and design, excluding an "understanding of planning laws and regulations", they showed relatively limited growth in understanding after training. The younger participants (20–39 years old) had lower planning awareness at the beginning, however the effect of training was better.

#### 3.2.2. Time Ratio of Living in the Community

The workshop participants did not permanently live in the community due to work commitments. Many of these participants can only return to the aboriginal community to reunite with family members during holidays. The proportion of time spent in the aboriginal community will also affect citizens’ perceptions and attitudes towards community planning.

**Figure 5:**
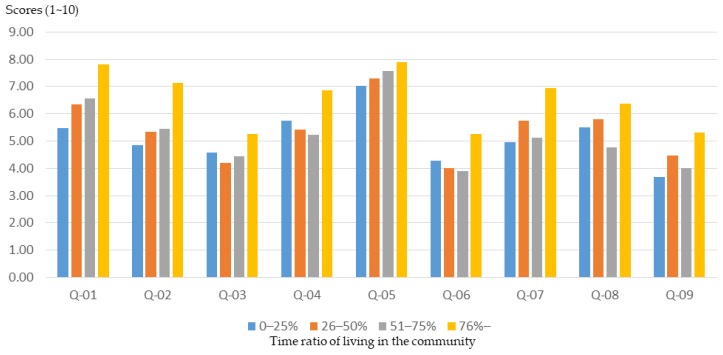
Workshop participants’ basic understanding before training with different time ratios of living in the community.

**Figure 6:**
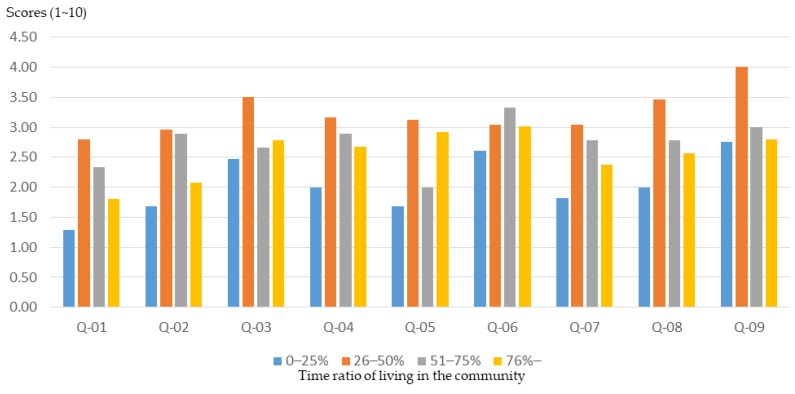
Basic cognitive growth of workshop participants after training, with different time ratios of living in the community.

**Figure 7:**
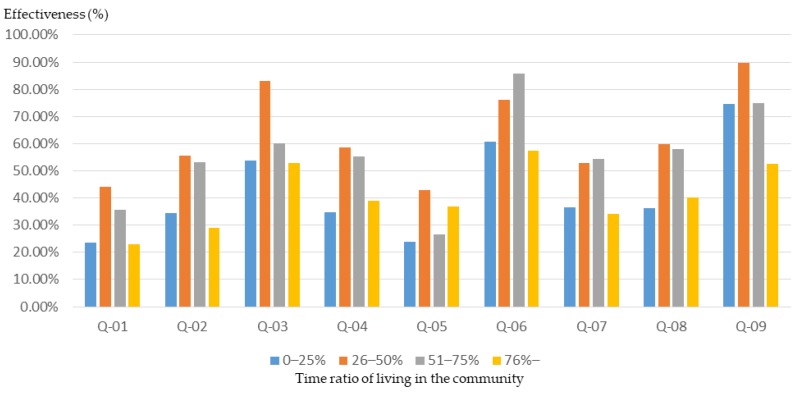
Basic cognitive growth rate of workshop participants after training, with different time ratios of living in the community.

In general, participants with a higher than 75%-time ratio of living in the community had higher initial basic knowledge about community planning (Figure 5) and were able to achieve higher training outcomes through workshops (Figure 6). However, participants with a lower time ratio of living in the community (0%–25%) were less understanding of community planning (Figure 5) and had relatively low training outcomes through workshops (Figure 7).

The results of this study show that participants with a higher time ratio of living in the community were able to enhance their basic understanding of planning and community awareness through planning training. In particular, in the three aspects of “understanding of spatial planning”, “understanding of planning laws and regulations”, and “understanding of GIS, GPS, and spatial analysis”, the workshop had a very significant effect on enhancing the understanding of participants.

#### 3.2.3. Educational Background

The educational background of the participants affects the training outcomes of the workshops and the effectiveness of the participatory planning. In order to understand the relationship between the educational background of participants and their basic planning cognition, this study conducted a relevant analysis.

**Figure 8:**
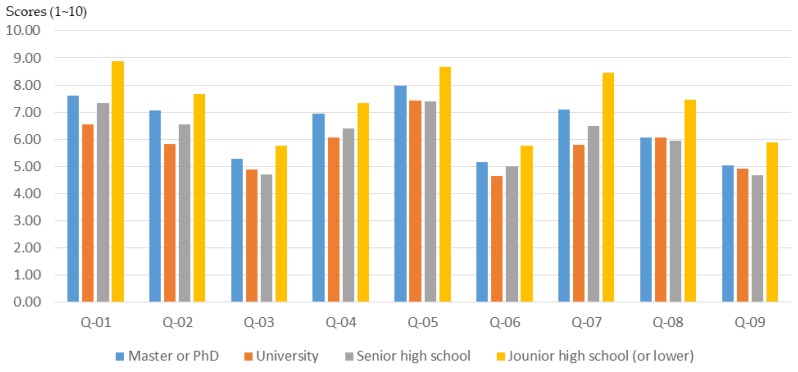
Workshop participants’ basic understanding before training, for different educational backgrounds.

**Figure 9:**
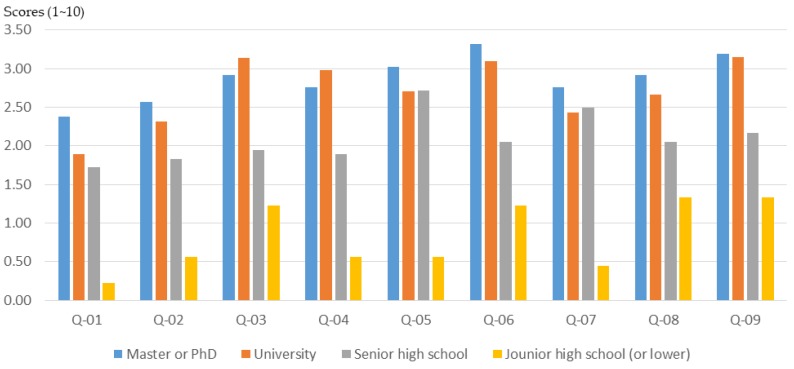
Basic cognitive growth of workshop participants after training, for different educational backgrounds.

**Figure 10:**
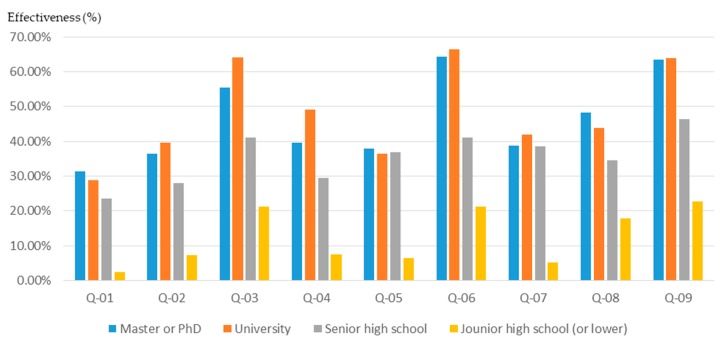
Basic cognitive growth rate of workshop participants after training, for different educational backgrounds.

The results of the study show that participants with a bachelor’s degree or above may have a lower initial knowledge of planning and design (Figure 8). However, after planning training in the workshop, their basic understanding of planning grew significantly (Figure 9 and Figure 10). In particular, in the three areas of “understanding of spatial planning”, “understanding of planning laws and regulations”, and “understanding of GIS, GPS, and spatial analysis”, the workshop achieved remarkable results in improving the basic understanding of planning.

Basically, citizens who have a junior high school educational background are usually older than 65 years old. The results of this study show that participants with an educational background of junior high school or lower experienced a lower training effect through the workshop (Figure 9; Figure 10). This result should be influenced by both the age and educational background of the participants.

#### 3.2.4. Roles in the Community

**Figure 11:**
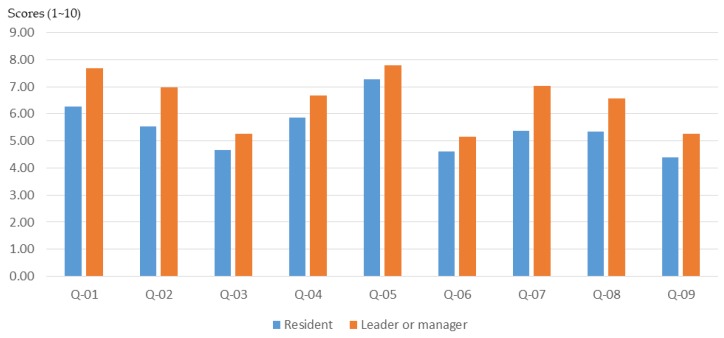
Workshop participants’ basic understanding before training, for different roles in the community.

**Figure 12:**
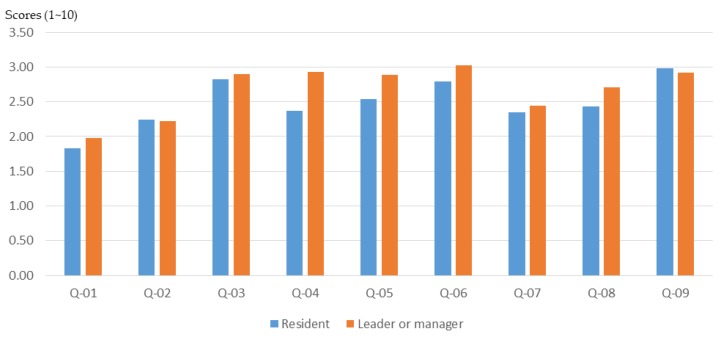
Basic cognitive growth of workshop participants after training, for different roles in the community.

**Figure 13:**
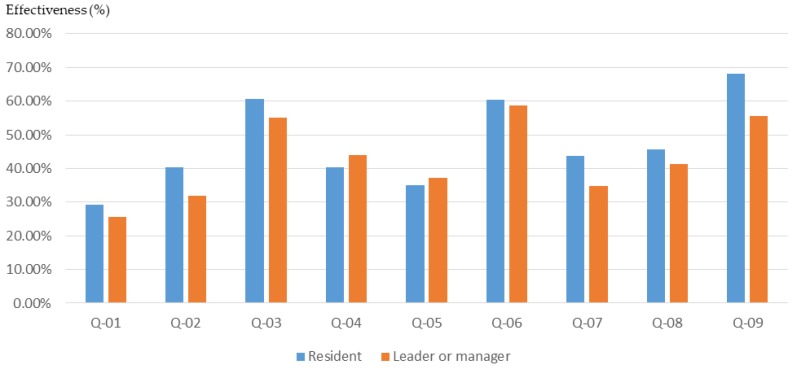
Basic cognitive growth rate of workshop participants after training, for different roles in the community.

The role played in the community (manager or general public) often determines a person’s views and opinions about issues. According to the results of this study, the participants with the role of community leader or community manager had a higher basic understanding of planning (Figure 11) and also higher cognitive growth after training through the workshop (Figure 12 and Figure 13).

Due to the low initial cognition and the significant effect of the workshop, there was a high rate of basic cognitive growth in the general public. The above research results show that due to the occupation of different roles in the community, the participants had different levels of understanding of the basic knowledge of the planning. Therefore, the training content of the participatory planning workshop should be divided according to the background of different participants in order to improve the training effect.

#### 3.2.5. Community Participation Experiences

**Figure 14:**
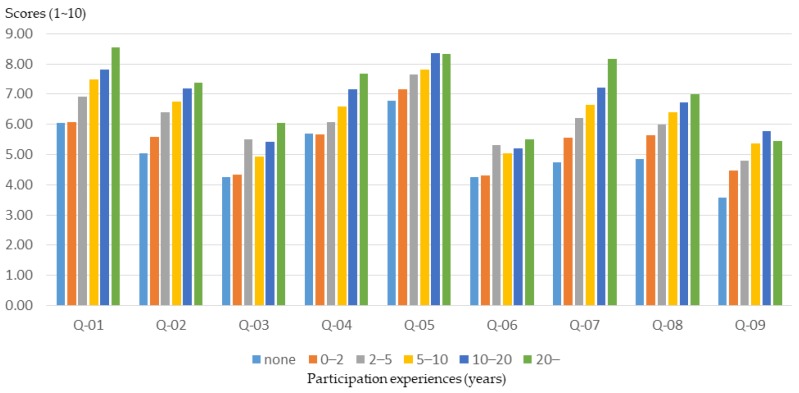
Workshop participants’ basic understanding before training, for different levels of community participation experience.

**Figure 15:**
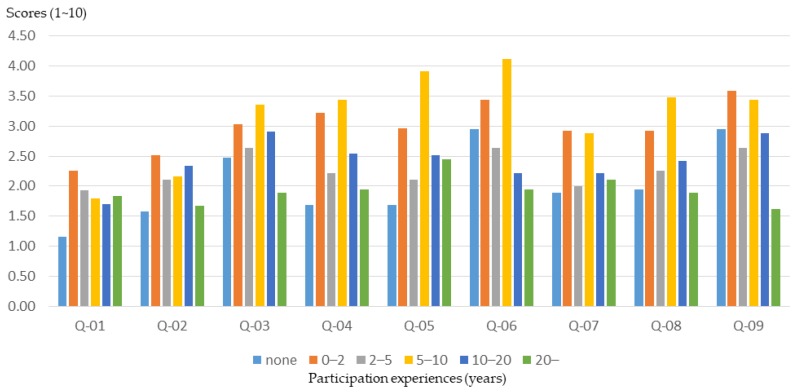
Basic cognitive growth of workshop participants after training, for different levels of community participation experience.

**Figure 16:**
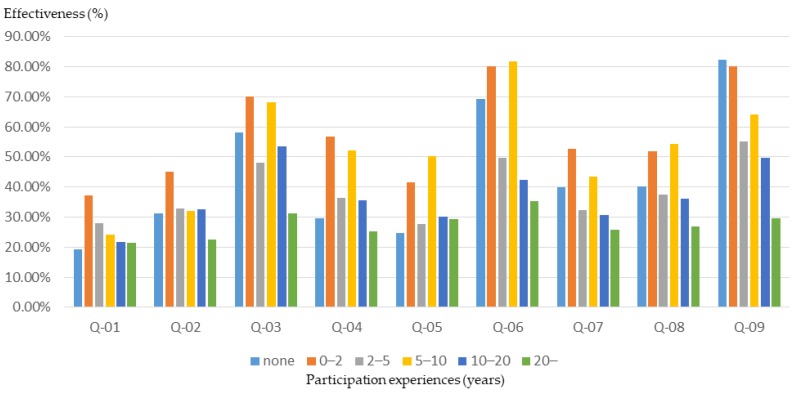
Basic cognitive growth rate of workshop participants after training, for different levels of community participation experience.

Differences in experience in participating in community public affairs often lead to different views and opinions on issues. In this study, the participants were grouped on the basis of community participation experience, and the relationship between participants’ community participation experiences and training effectiveness was clarified. According to the results of this study, participants with more community participation experience had a better understanding of the basics of planning (Figure 14). However, the participants with less community participation experience (0–10 years of qualifications) experienced a higher training effect (Figure 15 and Figure 16).

This result shows that the planning training produced a better basic learning effect for the general public. Therefore, for participants who have been involved in community affairs for many years (i.e., who already have a basic knowledge), event planners should provide advanced training courses and activities to improve the training outcomes.

### 3.3. Differences in Training Effectiveness among Participants from Different Tribes

**Figure 17:**
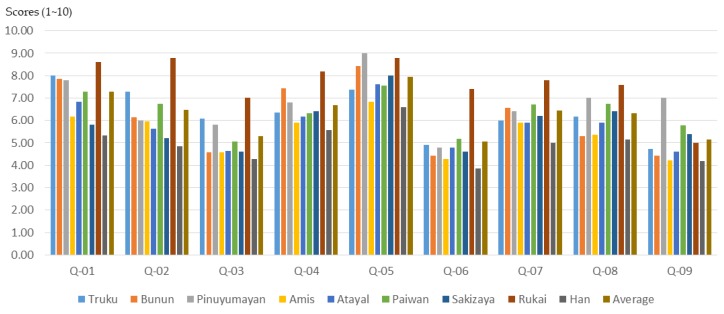
The basic understanding of participants from different indigenous tribes before training.

**Figure 18:**
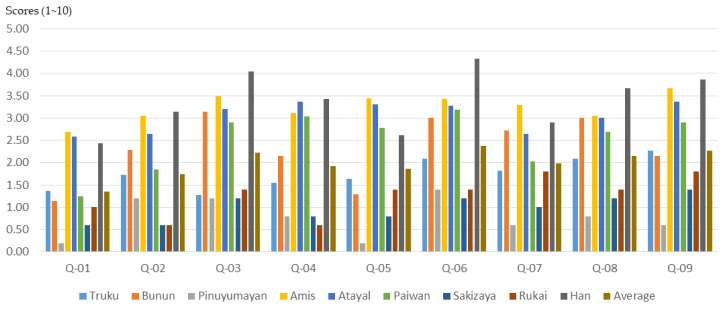
The basic cognitive growth of participants from different indigenous tribes after training.

**Figure 19:**
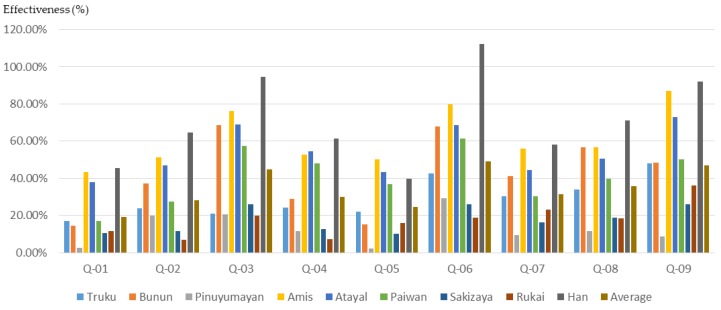
The basic cognitive growth rate of participants from different indigenous tribes after training.

The cultural background of the participants will affect the effectiveness of the participatory planning workshop and planning training. According to the results of this study (Figure 17, Figure 18 and Figure 19), participants from different indigenous tribes experienced different training effects for the same training course. Except for 21 non-indigenous people (Han), the participants sampled in this study belonged to eight Taiwanese indigenous tribes, namely Truku, Bunun, Pinuyumayan, Amis, Atayal, Paiwan, Sakizaya, and Rukai. Since the participants in the workshop either came from the government’s recommendation to participate, or signed up for themselves, this study could not conduct a proportional sampling survey based on the number of ethnic groups in Taiwan. Therefore, this study only analyzed the training benefits of all participants in the self-evaluation data before and after planning training.

#### 3.3.1. Truku

The Truku tribe has about 24,000 people, and has expertise in hunting, weaving, and knitting. The analysis of the basic cognitive and planning training effectiveness of the indigenous tribes showed that the Truku participants have high awareness of the “community environment”, “community planning”, and “the sense of community identity”. Additionally, Truku participants also have a good “understanding of spatial planning”, “understanding of the relationship between aboriginal ecological knowledge and community planning”, “understanding of the vision of the community’s planning”, and “understanding of the relationship between related plans and community planning”.

However, after the planning training, the Truku participants had substantially enhanced the cognitive standards of their “understanding of planning laws and regulations”, “understanding of the relationship between related plans and community planning”, and “understanding of GIS, GPS, and spatial analysis”. This result shows that, for the Truku participants who have a high level of community planning knowledge, planning training can enhance the functions of legal knowledge and spatial planning skills.

#### 3.3.2. Bunun

The Bunun tribe has a total population of about 50,000, and its family members may include non-family blood relations; therefore, Bunun traditional family residence usually has a large size. The highest awareness of the Bunun participants regarding community planning were in the three aspects of “community environment”, “the relationship between aboriginal ecological knowledge and community planning”, and “community identity”. The weaker areas of the Bunun participants’ awareness included the aspects of “understanding of spatial planning”, “understanding of planning laws and regulations”, and “understanding of GIS, GPS, and spatial analysis”.

After the planning training, the Bunun participants had the most significant recognition of the four aspects of “understanding of spatial planning”, “understanding of planning laws and regulations”, “understanding of the relationship between aboriginal ecological knowledge and community planning”, and “understanding of GIS, GPS, and spatial analysis”. This result shows that through the planning training, the cognitive improvement and skill enhancement of the Bunun participants with respect to their “understanding of spatial planning”, “understanding of planning laws and regulations”, and “understanding of GIS, GPS, and spatial analysis” were significant.

#### 3.3.3. Pinuyumayan

The Pinuyumayan tribe has a total population of about 11,000. At the peak of their military conquest, the Pinuyumayan tribe ruled over 72 aboriginal communities in the eastern part of Taiwan. The highest awareness of the Pinuyumayan participants regarding community planning were in the aspects of “community environment”, “community identity”, “spatial planning”, and “GIS, GPS, and spatial analysis”.

In addition to the understanding of planning laws and regulations, Pinuyumayan participants have a certain level of basic knowledge. However, because the basic knowledge of community planning has reached a certain level, the degree of cognitive growth obtained after planning training is limited. Therefore, it is very important to improve the training outcomes of participants who have a higher level of cognition through more advance training courses and activities.

#### 3.3.4. Amis

The Amis tribe has a total population of about 177,000 (the largest of Taiwan’s indigenous tribes), and their traditional social organization is based on a matrilineal system. Compared with the Pinuyumayan participants, the situation of Amis participants was slightly different. The Amis participants had a low understanding of all community planning basic knowledge. The aspects of which the Amis participants had a relatively higher understanding were “community environment” and “community identity”. The aspects of community planning of which the Amis participants had a relatively lower basic understanding were “understanding of spatial planning”, “understanding of planning laws and regulations”, and “understanding of GIS, GPS, and spatial analysis”.

Due to the low initial knowledge of community planning, after the planning and learning training, the Amis participants had experienced significant cognitive growth in “community planning”. Relatively higher cognitive growth rates were experienced in the “understanding of spatial planning”, “understanding of planning laws and regulations”, and “understanding of GIS, GPS, and spatial analysis” items.

#### 3.3.5. Atayal

The population of the Atayal tribe is approximately 81,000. The tribe has a traditional lifestyle involving farming and hunting, and has developed intricate fabric weaving skills, featuring sophisticated patterns and designs. For the Atayal, ancestral spirit worship is an important organized social activity, and the worship rituals constitute the major religious ceremony.

For Atayal participants, the highest awareness of community planning was in “community identity”. Atayal participants also had a high level of awareness of “understanding of the community environment” and “understanding of the relationship between aboriginal ecological knowledge and community planning”, and a low level of awareness of “understanding of spatial planning“, “understanding of planning laws and regulations” and “understanding of GIS, GPS, and spatial analysis”.

After the planning and learning training, the Atayal participants experienced significant growth in their basic understanding of community planning, especially in the three concepts of “understanding of spatial planning”, “understanding of planning laws and regulations”, and “understanding of GIS, GPS, and spatial analysis”. Specifically, the training results of the Atayal participants are similar to those of the Amis participants, since their initial basic cognition was low. For the Atayal participants, the benefits of planning training can be achieved for “understanding of spatial planning” and “understanding of GIS, GPS, and spatial analysis”, which is difficult for ordinary people to have the opportunity to contact or understand.

#### 3.3.6. Paiwan

The population of the Paiwan tribe is close to 86,000. The chief of each tribe is the leader in politics, military, and religion. In the Paiwan tribe, an alliance between close relatives is common among the noble families.

For Paiwan participants, the highest level of basic cognition was in the “understanding of the community environment” and “the sense of community identity” items. However, the cognition of the basic concepts of the “understanding of spatial planning” and the “understanding of planning laws and regulations“ were relatively low. However, unlike other ethnic groups, after planning training, the cognition of Paiwan participants mostly grew, especially in the “understanding of spatial planning”, “understanding of the relationship between aboriginal ecological knowledge and community planning”, “understanding of planning laws and regulations”, and “understanding of GIS, GPS, and spatial analysis” items.

#### 3.3.7. Sakizaya

The Sakizaya tribe, with a population of about 330, is based mainly on a matrilineal system, and has a traditional lifestyle involving fishing and hunting. For Sakizaya participants, the highest basic understanding was in the “sense of community identity” item, and the two lowest basic understandings were in the “understanding of spatial planning” and “understanding of planning laws and regulations” items. Their basic cognitive values for other community planning items were relatively low.

After planning training, the results for the Sakizaya participants were different from those for the Amis participants. The cognitive improvement of Sakizaya participants in various aspects of community planning was obviously limited. Even in the “understanding of spatial planning”, “understanding of planning laws and regulations”, and “understanding of GIS, GPS, and spatial analysis”, the community planning cognitive growth was only small.

#### 3.3.8. Rukai

The population of the Rukai tribe is about 11,600. The tribe has two hierarchy systems—the nobles and the commoners. The nobles have privileges conferred by bloodline “superiority” as found in the Rukai mythology. Common Rukai people can elevate their social status by developing individual leadership, increasing harvest production, or through marriage. 

Among the Rukai participants, the highest awareness of items of community planning were in the “understanding of the community environment”, “understanding of community planning”, “understanding of the relationship between aboriginal ecological knowledge and community planning”, and “the sense of community identity”. In addition to “understanding of GIS, GPS, and spatial analysis”, Rukai participants had a relatively high basic understanding of other items. However, after the planning training, aside from the specific improvement in the community planning cognition value of the “understanding of GIS, GPS, and spatial analysis”, the cognitive improvement of other community planning was limited. Therefore, this result shows that the content of the planning training should be adjusted for the characteristics of the Sakizaya participants and the Rukai participants to ensure the effectiveness of the training.

## 4. Conclusions

Since the 1970s, there have been practical cases in the United States for the participation of the public in the planning process [35]. The participation of citizens in the planning process can be seen not only as citizens’ power [36], but also as a practice of “theories of collective intelligence and crowd wisdom” [37]. Through the participation of citizens in the planning process, the government and planning professionals can understand the real life needs of the community and the opinions on the planning plan, and can also reduce the public resistance to planning. Citizens can share local life experiences, cultural knowledge, and traditional wisdom in the participatory planning process. Furthermore, through discussions with the public, planners can predict the conflicts between economic development, ecological conservation, and quality of life, and can work with the public to propose solutions. For instance, there are many indigenous communities in Taiwan located in popular tourist and leisure areas. The discussion of the public participation mechanism is helpful to achieve balance between ecological protection and tourism industry development [38].

However, in order to make participatory planning more effective, people must have some basic knowledge of concepts related to planning before they actually participate in the planning process. Therefore, planning training is important for citizen participation in the planning process. However, with the differences in participants’ age, education level, community participation experiences, cultural background, etc., the effect of planning training is also different.

According to the results of this study, compared to young (20–49 years old) participants, the middle-aged participants (50–59 years old) became more proficient in planning-related concepts and understanding after training courses and activities. Additionally, although older participants (over 60 years of age) had a higher initial understanding of planning and design, they experienced relatively limited cognitive growth after training. These results indicate that the content of planning training courses and activities must be adjusted for participants of different age groups. However, some activities must also be planned to allow participants of different ages to have opportunities for interaction and discussion to eliminate objections and gaps between generations.

The results of this study show that participants with a higher time ratio of living in the community were able to enhance their understanding of basic concepts of planning and community awareness through planning training. However, participants with a lower time ratio of living in the community (0%–25%) not only had a lower basic understanding about community planning, but also had relatively poor training outcomes through workshops. In fact, there are many people in Taiwan who have left their hometowns to work in metropolises. These people cannot live in small communities for a long time, so they often lack a basic understanding of concepts of the community and community planning. However, everyone involved in planning training has the enthusiasm of community planning. The organizers of planning training courses and activities should provide special training courses for people who cannot live in a community for a long time to enhance the training effect.

The educational background of participants will affect the training outcomes of a workshop and affect the effectiveness of the participatory planning activities. The results of this study show that participants with a bachelor’s degree or above may have a lower initial knowledge of planning and design. Nevertheless, after planning training in the workshop, their basic understanding of planning grew significantly. In Taiwan, it is very common for people to have a high level of education, with people having junior high school education or lower usually being older than 65 years old. The results of this study show that participants with an educational background of junior high school or lower have a lower training effect through the planning training. Therefore, in order to enhance the training effectiveness, there must be some special planning training courses for participants with low educational attainment.

The role played in the community and the community participation experiences are two important factors influencing the community’s awareness and the understanding of community planning concepts. According to the results of this research and analysis, the workshop participants who occupy the role of leader or manager of their community have a higher basic understanding of planning and also have higher cognitive growth after planning training. However, the training effect on some participants who have been involved in community affairs for many years (who already have a basic knowledge) is significantly low in this study. Therefore, in order to enhance the overall effect of planning training, the organizers of planning training courses and activities should provide different levels of courses and activities for different levels of participants, especially for those who have more experience in community public affairs and leaders or managers of community organizations.

The cultural background of the participants affects the effectiveness of citizen participatory planning and planning training. According to the results of this study, participants from different aboriginal ethnic groups experience different training effects following the same training course; some aboriginal participants receive little training effect, since the same training courses and activities are not suitable for every participant. Governments and planners should develop exclusive planning training courses and activities based on different community environments and different aboriginal cultural backgrounds to ensure the effectiveness of planning training and citizen participatory planning.

In addition, although this study belongs to the local case study in Taiwan, the research results of planning training to enhance the efficiency of participatory planning in this research are quite valuable in the international related field. Therefore, the results of this study can provide a reference for governments and planning professionals to formulate planning training courses and activities and citizen participatory planning in the future, as well as a reference for subsequent researchers. Besides, the discussion of what levels of additional training would be required in order to empower stakeholders to engage in planning decision-making at the higher levels of the ladder of participation is very important. Since this part is not covered in this study, it is recommended that related research should be conducted to explore such aspects.

## Figures and Tables

**Table 1:** Expert background information from the expert questionnaire.

Topics	Contents	Hours
Planning Theory	1. Indigenous peoples and spatial planning	1.5
	2. The basic concept of planning	1.0
	3. Planning related laws and regulations	1.5
	4. Indigenous peoples’ culture of land and space	1.5
Tools and Training	1. People’s participation in spatial planning	1.5
	2. Substantial planning: indigenous community planning	1.0
	3. Map, GIS, information interpretation, and spatial analysis	1.5
	4. Community Survey: citizen participation	1.5
Practice & Discussion	1. Community planning case study	1.0
	2. Comprehensive discussion	0.5
Total Hours:		12.5

**Table 2:** The questionnaire content of the workshop.

Question Number	Topics
Q-01	Understanding of the community environment
Q-02	Understanding of community planning
Q-03	Understanding of spatial planning
Q-04	Understanding of the relationship between aboriginal ecological knowledge and community planning
Q-05	The sense of community identity
Q-06	Understanding of planning laws and regulations
Q-07	Understanding of the vision of the community’s planning
Q-08	Understanding of the relationship between related plans and community planning
Q-09	Understanding of GIS, GPS, and spatial analysis

**Table 3:** Background information of 167 participants.

Tribe	People	Age	People	Time Ratio of Living in the Community (%)	People	Educational Background	People	Roles	People	Community Participation Experience (years)	People
Truku	11	20–29	43	0–25%	37	PhD or master’s	45	Citizen	67	0	26
Bunun	7	30–39	46	26–50%	25	University	95	Leader	77	0–2	25
Pinuyumayan	5	40–49	33	51–75%	10	Senior high school	15	Unanswered	23	2–5	29
Amis	45	50–59	23	≥76%	82	Junior high school (below)	8			5–10	28
Atayal	36	60–69	14	Unanswered	13	Unanswered	4			10–20	29
Paiwan	32	70–	4							≥20	15
Sakizaya	5	Unanswered	4							Unanswered	15
Rukai	5										
Han (non-indigenous)	21										
